# Trans-visceral migration of retained surgical gauze as a cause of intestinal obstruction: a case report

**DOI:** 10.1186/1752-1947-2-17

**Published:** 2008-01-24

**Authors:** Nello Grassi, Calogero Cipolla, Adriana Torcivia, Alessandro Bottino, Eugenio Fiorentino, Leonardo Ficano, Gianni Pantuso

**Affiliations:** 1 Department of Oncology – Division of General and Oncological Surgery, University of Palermo, Palermo, Italy

## Abstract

**Introduction:**

A retained surgical sponge in the abdomen is uncommon although it is likely that this finding is underreported in the medical literature. The intravisceral migration of retained surgical gauze is even rarer, as demonstrated by the very few cases reported.

**Case presentation:**

Three years after undergoing anterior resection of the rectum, a 75-year-old man presented with symptoms of small bowel obstruction. Plain abdominal radiography and CT showed a radio-opaque marker; a foreign body was suspected, probably a piece of retained surgical gauze. An ileotomy of about 5 cm. was performed to confirm this diagnosis and remove the gauze.

**Conclusion:**

Although rare, retained gauze in the abdomen is a complication of surgery. The authors consider that this event may be more frequent than it appears from reports in the literature, probably because of its medico-legal implications. If all such cases were reported, it would be possible to estimate their exact number, classify the occurrence as a possible surgical complication and thus modify its medico-forensic consequences.

## Introduction

Retained surgical gauze in the abdominal cavity is an infrequent event but it may cause symptoms, both in the early postoperative period as well as months or years after the original operation. Non-specific clinical symptoms and inconclusive imaging findings may preclude an accurate diagnosis [1]. It can, however, be diagnosed preoperatively in many instances with the help of radiological studies such as plain radiography, when surgical textile materials have been impregnated with a radio-opaque marker. Ultrasonography (USG), computerized tomography (CT), magnetic resonance imaging (MRI), and gastrointestinal contrast series [2,3] can all be used to assist in diagnosis. A surgical sponge may completely migrate into the intestinal lumen without any apparent opening in the intestinal wall [4]. We report a case of retained surgical gauze causing an intestinal obstruction due to its migration into the small bowel; to the best of our knowledge, there are few similar cases reported in the medical literature [4,5].

## Case presentation

**Figure 1:**
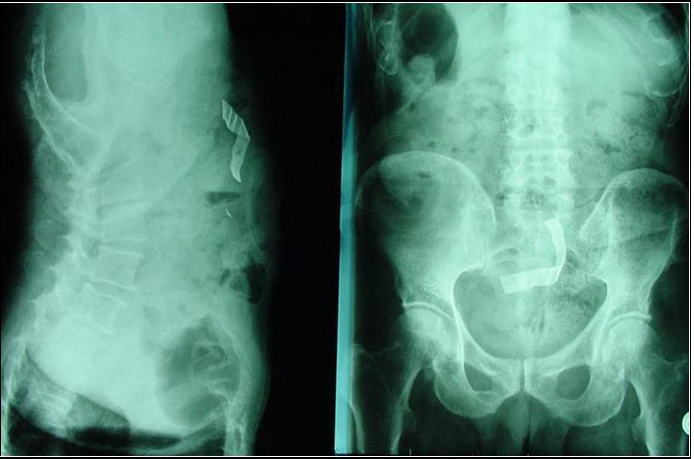
Plain abdominal radiography.

**Figure 2:**
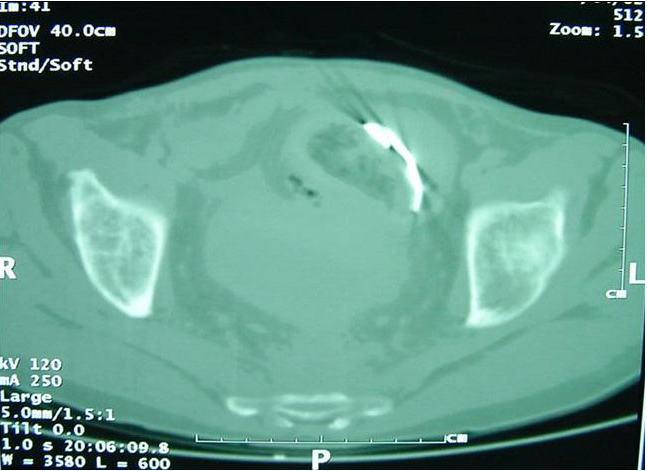
Contrast enhanced abdominal CT.

A 75-year-old man, who had undergone anterior resection of the rectum in our department three years previously for rectal carcinoma, was readmitted into our surgical unit because of a series of symptoms including abdominal colic, pain in the epigastrium, nausea, alvus closed to faeces but not to gas and fever (38.5°C). Clinical examination revealed abdominal tension, with intense pain on palpation in the epigastric region and in the right iliac fossa. Whole blood analysis showed a marked leukocytosis (white blood cells: 23.200/mm3) while the other biochemical parameters were within normal limits. Plain abdominal radiography (figure 1) and successive contrast-enhanced abdominal CT (figure 2) revealed marked dilatation of the proximal jejunal segments, hydro-aerial levels on in the left abdominal quadrants and a radio-opaque foreign body of about 5 centimeters in diameter, contiguous to an ileal ansa and quite free within the pelvic cavity. The presence of a foreign body, possibly a piece of retained surgical gauze, was considered.

**Figure 3:**
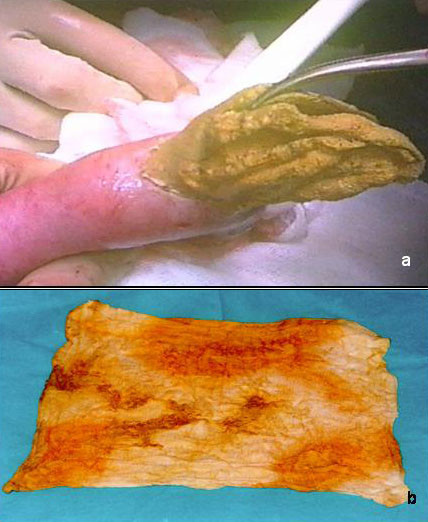
a) Removal of laparotomic gauze across ileal breach. b)Laparotomic gauze 40 × 40 cm.

We performed a xifo-pubic laparotomy and, after detaching several dense adhesions, observed that the final ileal ansa was deformed by the presence in its lumen of a pulpy, ovaliform structure, 10 cm in width. No signs of previous fistulas or abscesses of the intestinal wall were detected. An ileotomy of about 5 cm made it possible to remove the foreign body, which proved to be a piece of laparotomic gauze of 40 × 40 centimeters (figure 3). The ileal breach was then closed with interrupted PDS 2/0 sutures. After a brief and uneventful post-operative period, the patient was discharged in good general health.

## Discussion

A gauze swab left in the abdomen represents an extremely serious iatrogenic complication in major abdominal laparotomic surgery. The most frequent (69%) type of foreign body left inside the abdomen during surgery is, in fact, laparotomic gauze. Several factors which may make it necessary to cut down on operating time may lead to a consequent increase in the risk ratio of such an occurrence by from 1,1 to 8,8; these include not only emergency surgery, but also a high body mass index of the patient and intraoperative complications, such as hemorrhage [6].

Several measures might help to avoid the problem of leaving foreign bodies inside the abdomen, especially in the case of high-risk patients, for example, an accurate swab count prior to surgery, during the operation and finally after the incision has been closed. Further measures include the use of radio-opaque sponges only and a careful control of the inside of the abdomen by the surgeon before closure. Moreover, if there is any doubt about the accuracy of the final swab count, intraoperative radiologic screening may detect any retained surgical material impregnated with a radio-opaque marker.

It is not easy to say whether cases of gauze left in the abdomen are always due to a real lack of quality on the part of the surgeon or of the theater nurse. Most retained sponges, in fact, occur after "normal" swab counts, perhaps falling outside the human safeguards designed to prevent these types of errors [6].

Clinical symptoms may be various and closely connected to the subsequent destination of the textile aid. Abscess formation with peritonitis or systemic septic symptoms has been reported to occur in 16.7% of the cases, hemoperitoneum secondary to a vessel lesion or an abdominal mass with no apparent symptoms in 8.3% and intestinal obstruction in 58.3% of the cases. Moreover it has been reported that the interval between the probable causative operation and the diagnosis of retained gauze may range from 11 days to 28 years [7].

It is extremely difficult to reach a correct diagnosis of retained surgical gauze and in particular of any transvisceral migration by means of clinical examination only. Instrumental examinations, such as plain abdominal radiography, which will show a radio-opaque marker, and abdominal USG and contrast-enhanced abdominal CT in order to show up the mass image, are generally essential [8], even though preoperative imaging studies [4,5] are able to reveal the exact site of migration of a retained surgical textile aid into the intestinal lumen in very few cases. This was also the case for our reported patient.

Although a surgical sponge may migrate completely into the ileum without any apparent opening in the intestinal wall [4], various hypotheses have been formulated to explain exactly how the presumed transvisceral migration of such a foreign body might occur, and, moreover, without causing any particular parietal alteration. Dhillon and Park [5] have suggested that the presence of gauze might bring about an inflammatory reaction surrounded by a subsequent abscess pouch, followed by intestinal perforation and sponge migration into the lumen of the small bowel.

Even though prevention is the best treatment as in many other medical problems, the only considered therapy for a retained surgical swab in the abdomen remains surgical removal, which may lead to a 10% mortality rate if there is delay after diagnosis [8]. In fact, even though alternative methods such as percutaneuos extraction have been proposed [9], we believe that these minimally invasive methods are not very useful for the removal of foreign bodies from the abdomen, in particular because laparotomy may reveal several dense adhesions between the foreign body and intra-abdominal organs.

The incidence of the phenomenon "retained textile aid" during surgical procedure, reported in several case reports, is of about 1/1000–1500 procedures on average [5]. Kaiser et al [10], revising the Medical Professional Insurance Company Archives of Boston, noticed that a falsely correct gauze count happens in 76% of the cases where legal procedure had been undertaken for a retained sponge in the abdomen. Nevertheless, since this figure derives mainly from forensic literature or from the registers of insurance companies involved in legal compensation for malpractice, it may well be that it does not reflect the real incidence of the phenomenon. We ourselves maintain that if all such cases were openly reported, the incidence would most certainly be higher and could be listed among the other possible surgical complications, which are impossible to eliminate completely, and that this could lead to a considerable change in medico-forensic attitudes towards the problem.

Nevertheless, in spite of continual improvement in surgical procedures and the technical evolution aimed at protecting patients in the operating theatre, published data report that the problem of residual foreign bodies after surgery is still unresolved and, furthermore, the scarcity of reports regarding this event, probably due to the inevitable medico-forensic implications, means that its incidence is still underestimated.

It is therefore to be hoped that cases of retained surgical gauze in the abdomen will be constantly reported in the medical literature in future, in order to make a real estimate of the incidence of this event, to standardize recommended procedures for avoiding it, but above all, in order to modify the medico-forensic implications of the phenomenon.

## Competing interests

The author(s) declare that they have no competing interests.

## Authors' contributions

Grassi N., Torcivia A. and Bottino A. performed the operation, assessed the didactic importance of the clinical case and acquired the data; Cipolla C. Pantuso G. and Torcivia A. were responsible for drafting the manuscript and its revision; Fiorentino E. and Ficano L gave their final approval of the version to be published. All Authors read and approved the final manuscript.

## Consent

Written informed consent was obtained from the patient for publication of this Case report and any accompanying images. A copy of the written consent is available for review by the Editor-in-Chief of this Journal.
